# MGACL: Prediction Drug–Protein Interaction Based on Meta-Graph Association-Aware Contrastive Learning

**DOI:** 10.3390/biom14101267

**Published:** 2024-10-08

**Authors:** Pinglu Zhang, Peng Lin, Dehai Li, Wanchun Wang, Xin Qi, Jing Li, Jianshe Xiong

**Affiliations:** 1 Faculty of Information Science and Engineering, Ocean University of China, Qingdao 266003, China; zpl1668@stu.ouc.edu.cn (P.Z.); vanchun@stu.ouc.edu.cn (W.W.); 2 Key Laboratory of Marine Drugs, Chinese Ministry of Education, School of Medicine and Pharmacy, Ocean University of China, Qingdao 266003, China; plin.r@foxmail.com (P.L.); dehaili@ouc.edu.cn (D.L.); qixin_ouc@ouc.edu.cn (X.Q.)

**Keywords:** drug–target interaction, graph neural networks, contrastive learning, heterogeneous graph representation

## Abstract

The identification of drug–target interaction (DTI) is crucial for drug discovery. However, how to reduce the graph neural network’s false positives due to its bias and negative transfer in the original bipartite graph remains to be clarified. Considering that the impact of heterogeneous auxiliary information on DTI varies depending on the drug and target, we established an adaptive enhanced personalized meta-knowledge transfer network named **M**eta **G**raph **A**ssociation-Aware **C**ontrastive **L**earning (MGACL), which can transfer personalized heterogeneous auxiliary information from different nodes and reduce data bias. Meanwhile, we propose a novel DTI association-aware contrastive learning strategy that aligns high-frequency drug representations with learned auxiliary graph representations to prevent negative transfer. Our study improves the DTI prediction performance by about 3%, evaluated by analyzing the area under the curve (AUC) and area under the precision–recall curve (AUPRC) compared with existing methods, which is more conducive to accurately identifying drug targets for the development of new drugs.

## 1. Introduction

The increasing gap between drug development speed and treatment demand drives researchers to enhance research efficiency and reduce costs [1]. Drug targeting is crucial for drug discovery [2], cutting down research time and expenses by repurposing existing drugs. Drug–target interaction (DTI) prediction is vital in this process, aiding in the identification of potential drug targets and the design of more effective and safer therapies. In 2023, the FDA approved 55 innovative treatments, a nearly 50% increase from 2022 [3], highlighting technological advancements in the field. Thus, DTI prediction is essential not only for drug discovery but also for drug repositioning and personalized medicine development.

The massive accumulation of histological data provides new prospects for DTI prediction research. DTI prediction has gradually evolved from traditional biological experiment-based methods to machine learning-based methods [4,5]. Machine learning-based methods can be subdivided into feature-based methods, similarity-based methods, and network-based methods. For example, Zhao et al. [6] proposed HyperAttentionDTI, which innovatively uses one-dimensional convolutional layers to enhance feature representation and obtain potential DTIs. Similarity-based methods often follow the “guilt-by-association” principle, which assumes that similar targets often interact with similar drugs and vice versa. Liu et al. [7] proposed a fine-grained selective similarity integration method that adopts a weight matrix based on local interaction consistency to capture and utilize finer-grained similarities in the similarity selection and integration steps. A novel Multi-level Representation Learning Contrastive and Adversarial Learning (MRL_CAL) model was proposed [8], which estimates the distribution of the original incomplete data through adversarial learning and transforms incomplete multi-view clustering into an overall objective, allowing the model to learn features under the guidance of clustering. Based on incomplete interaction information, Yu et al. mapped all disease genes and drug genes onto a combined protein interaction network [9]. By calculating the module distance, they evaluated the distance between drug gene sets and disease gene sets, and thus inferred drug–disease associations. Similarly, to reduce drug development costs, weighted drug and disease networks have been constructed using drug side effects and disease symptoms [10]. These networks are then clustered into modules of related drugs and diseases, and drug–disease associations are ranked based on the connectivity between the modules.

Recent advances in graph neural networks (GNNs) [11,12] have enhanced the feature representation of entities and potential correlations in histological data, improving drug repositioning opportunities. Li et al. [13] proposed SGCL-DTI, which can generate a contrastive loss by comparing the topological structure and semantic features of the same graph and constrain the model to iterate continuously in a supervised manner. Zhang et al. [14] used contrastive learning to fuse different neighborhood features and finally obtain drugs, proteins, and common domain characteristics of diseases. To more accurately express interaction relationships, a model called joining Nonnegative Matrix Factorization and Graph Contrastive Learning (jNMF-GCL) was proposed [15]. This model performs contrastive learning by selecting positive and negative samples from a constructed affinity graph.

Although the performance is encouraging, the frequency distribution of drug nodes in interactive data is often uneven. Models trained on these imbalanced datasets are prone to overfit those “high-frequency” drugs or proteins, meaning that the models tend to predict interactions based on known interaction frequencies of drugs or targets while ignoring their actual biological compatibility [16]. This bias can even lead to the Matthew effect [17], which is a feedback loop where the rich get richer. In actual DTI prediction scenarios, we observe that when some less common drugs or targets receive sufficient research attention and data accumulation, their potential interactions can also be accurately identified and validated, indicating that not all of these less common drugs or targets are inefficient or irrelevant. If we can learn better representations of these relatively uncommon drugs or targets, we may discover and predict their potential effective interactions, thereby making more accurate predictions.

Following this line of thought, a simple idea is to provide more connections for the tail nodes. Although the feature data of tail nodes are usually sparse, in most cases, their attributes are as complete as those of the head nodes. Another method is to generate edges between nodes with some similarity, such as in [16]. However, this can lead to several issues: (1) The original feature dimensions are high, and there is sparsity, making it difficult to find two spatial points with the same features; (2) consistency with the original binary structure is not considered, which would result in sub-optimal predicted edges. Therefore, we suggest learning the relationships between nodes as auxiliary graphs to combine with the rest of graph-based prediction training and updating the graph structure based on node attributes. However, the introduction of auxiliary graphs in self-supervised learning may introduce noise into drug node representations that have been adequately represented in the context and lead to negative transfer. Therefore, the key challenges are (1) how to transmit the extracted local connectivity relationships between nodes in different graph structures in an unbiased manner and (2) how to avoid introducing noise and overfitting the downstream skewed data distribution when learning heterogeneous relationships based on the relation-aware topological structural signals of different drug nodes.

To address the above challenges, we propose a novel **M**eta **G**raph **A**ssociation-Aware **C**ontrastive **L**earning (MGACL) model for drug–target interaction prediction. Specifically, we utilize a heterogeneous graph neural network as an encoder, where heterogeneous graphs can represent complex relationships and diverse information, which is preserved in the embedding of the encoding structure. To cope with personalization enhancement, we propose a novel contrastive learning framework that can encode personalized features of extracted drugs and targets using meta-local connected knowledge transfer networks. This allows us to perform drug- and target-specific enhancements, imposing regularization constraints via meta-learning methods to transfer relation-aware signals between different graph structures. To solve the negative transfer problem, we propose a DTI association-aware contrastive learning strategy that can simultaneously learn information from both the auxiliary graph and the original drug–target bipartite graph, ensuring their consistency. We consider the drug node representations of the auxiliary graph and the original bipartite graph as two views of specific drug nodes. For those drug nodes with trustworthy representations in the original graph, we use a multi-view contrastive learning strategy to align their representations to the two graphs. For those drug nodes with fewer relation-aware signals, contrastive learning nearly stops, thus preventing learning additional noise from affecting the auxiliary graph.

The main contributions of this study are summarized as follows:

- MGACL prevents negative transfer through the DTI association-aware contrastive learning strategy and safely incorporates heterogeneous auxiliary information into the model under the graph contrastive learning paradigm.
- MGACL combines meta-knowledge transfer networks with novel contrastive learning strategies to transmit personalized signals between different relationship views.
- Extensive experiments on five datasets show that MGACL can significantly improve performance over other strong baselines. Moreover, we validated the effectiveness of MGACL prediction results using molecular docking, providing an effective strategy for identifying drug–target interactions.

## 2. Methods

**Figure 1:**
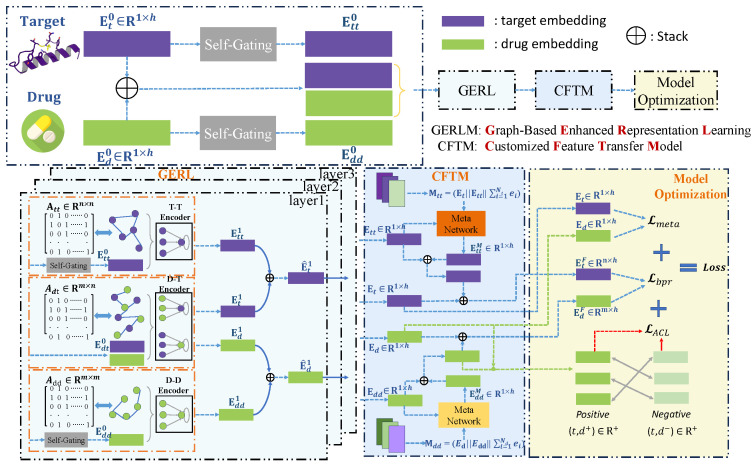
The framework of MGACL.

In this section, we introduce the framework of the MGACL model in detail. MAGCL mainly consists of three parts: (1) graph-based enhanced representation learning; (2) a customized feature transfer model; and (3) model optimization. Firstly, to represent the complex relationships and diverse information between drug–target, drug–drug, and target–target, graph-based enhanced representation learning is used to adaptively learn their correlations. Secondly, to address the issue of negative transfer, we propose a customized feature transfer strategy that can simultaneously learn information from both the auxiliary graph and the original drug–target bipartite graph, ensuring their consistency. Finally, using a two-stage loss function ensures that the model optimizes at multiple levels simultaneously, improving its ability to focus on optimizing predictive rankings and capture personalized knowledge and enabling the model to better distinguish between positive and negative samples. The overall framework of MGACL is shown in Figure 1.

### 2.1. Preliminaries

Drugs and targets in the real world are usually heterogeneous, containing different semantic information. We use the graph $G_{dt}=\left\{V_{d},V_{t},E_{dt}\right\}$ to represent the drug–target interaction relationship, where $V_{d}$ and $V_{t}$ represent the sets of drugs and targets, respectively. In $G_{dt}$, if there is a known interaction relationship between target *t* and drug *d*, then there is an edge $\left(\left(d,t\right)\in E_{dt}\right)$ between *d* and *t*. To better represent the local connectivity network between targets, graph $G_{tt}=\left\{V_{t},E_{tt}\right\}$ is defined as the set of $E_{tt}$ that includes the local connectivity relationships of targets. Define graph $G_{dd}=\left\{V_{d},E_{dd}\right\}$ as the local connectivity network between drugs. The auxiliary graph is constructed through the local connectivity of drug–drug and target–target relationships, and is aligned with the original drug–target interaction using a contrastive learning strategy to enhance the representation of sparse data nodes and improve the prediction performance of drug–target interactions. To prevent the learning auxiliary graph from being too dense and interfering with graph convolution, following [18], we threshold the sparsely learned locally connected relational network for interception, retaining only the first 5% edges. We define three adjacent matrices $A_{dt}\in R^{m\times n}$, $A_{dd}\in R^{m\times m}$, and $A_{tt}\in R^{n\times n}$, corresponding to graphs $G_{dt}$, $G_{dd}$, and $G_{tt}$, respectively. Here, *m* and *n*, respectively, represent the numbers of drugs and targets.

### 2.2. Graph-Based Enhanced Representation Learning

#### 2.2.1. Embedding Initialization with Relational Context Awareness

To encode heterogeneous relationships through high-order information modeling, we use a heterogeneous GNN to learn embeddings from drug–target graph $G_{dt}$, drug–drug graph $G_{dd}$, and target–target graph $G_{tt}$. First, we assign the corresponding embedding $e_{d},e_{t}\in R^{h}$ initialized by the Kaiming initializer [19], where *h* represents the hidden dimension. The initial embedding matrices $E_{d}^{0}\in R^{m\times h}$ and $E_{t}^{0}\in R^{n\times h}$ formed were specific for node embedding. To emphasize the differences in interaction patterns, we trained a self-gating [20] module to derive local connectivity relation-aware embeddings of drugs and proteins from a common initial embedding space, which can be determined as follows:

(1) $$\begin{array}{cc} & E_{dd}^{0}=E_{d}^{0}⊙\sigma \left(E_{d}^{0}W_{g}+b_{g}\right),\\ \; & E_{tt}^{0}=E_{t}^{0}⊙\sigma \left(E_{t}^{0}W_{g}+b_{g}\right), \end{array}$$

where $E_{dd}^{0}\in R^{m\times h}$ and $E_{tt}^{0}\in R^{n\times h}$ are embeddings of homogenous graphs $G_{dd}$ and $G_{tt}$ for drug–local connectivity relationships and protein–local connectivity relationships, respectively. To project the initial features into the embedding space, merge contextual relationships, and capture complex feature interactions and nonlinear relationships in the context, σ(·) is set as the sigmoid activation function. ⊙ represents the element-wise multiplication operation. $W_{g}\in R^{h\times h}$ and $b_{g}\in R^{h\times 1}$ are transformation and bias parameters [21].

#### 2.2.2. Heterogeneous Relation-Aware Signal Propagation

In the initial embedding matrix mentioned above, $E_{d}^{0}$ and $E_{t}^{0}$ are used as inputs for the drug–protein view, while $E_{dd}^{0}$ and $E_{tt}^{0}$ are used as inputs for the drug–drug local connectivity view and the protein–protein local connectivity view, respectively. First, the graph convolutional neural network (GCN) is used as the encoder of the three views; given an original bipartite graph $G_{dt}$, MGACL iteratively refines the drug and protein embedding via relation-aware signal propagation as follows:

(2) $$\begin{array}{cc} \; & e_{d}^{k+1}=\sum_{t\in N_{d}}\frac{1}{\sqrt{|N_{d}|}\sqrt{|N_{t}|}}e_{t}^{k},\\ \; & e_{t}^{k+1}=\sum_{d\in N_{t}}\frac{1}{\sqrt{|N_{t}|}\sqrt{|N_{d}|}}e_{d}^{k}, \end{array}$$

where $N_{d}$ and $N_{t}$ denote the neighbor sets of nodes *d* and *t*, respectively. $e_{d}^{k},e_{t}^{k}\in R^{h}$ denote the embedding vector of drug *d* and protein *t* in the k-th iteration. $e_{d}^{0}$ and $e_{t}^{0}$ are the row vectors of the embedding matrices $E_{d}^{0}$ and $E_{t}^{0}$, respectively. Inspired by a simplified convolution-based message-passing mechanism [22], using linear (average) aggregation ensures that information propagates smoothly and controllably, preventing embedding size from increasing with graph convolution operations. Similarly, the embedding $E_{dd}^{k}$ of the drug–drug local connectivity relation graph and the embedding $E_{tt}^{k}$ of the protein–protein local connectivity relation graph are iteratively refined according to the same GCN pattern.

#### 2.2.3. Message Integration in Heterogeneous Contexts

Inspired by the *soft meta-path* design in [23], drug and protein embeddings are updated through the heterogeneous fusion process defined below:

(3) $$\begin{array}{cc} \; & {\hat{E}}_{d}^{k+1}=f\left(E_{d}^{k+1},E_{dd}^{k+1}\right),\\ \; & {\hat{E}}_{t}^{k+1}=f\left(E_{t}^{k+1},E_{tt}^{k+1}\right), \end{array}$$

Here, the refined embedding in the *k* + 1st iteration ${\hat{E}}_{d}^{k+1}\in R^{m\times h}$, ${\hat{E}}_{t}^{k+1}\in R^{n\times h}$ merges rich semantic information and passes it on to the next layer for input. *f* represents the element-wise mean pooling function, which is used to perform fusion operations on heterogeneous information.

To further utilize the layer-specific representation of encoding (1≤k≤K) to aggregate isomeric information, we generate overall embeddings of drugs and proteins as follows:

(4) $$\begin{array}{cc} \; & E_{d}=E_{d}^{0}+\sum_{k=1}^{K}\frac{E_{d}^{k}}{∥E_{d}^{k}∥},\\ \; & E_{t}=E_{t}^{0}+\sum_{k=1}^{K}\frac{E_{t}^{k}}{∥E_{t}^{k}∥}, \end{array}$$

where *K* denotes the maximum number of GCN iterations. We use skip connections to add initial embeddings $E_{d}^{0}$ and $E_{t}^{0}$. Equation (4) indicates the layer-specific representation aggregation of the drug–protein interaction view. The embedding of drug–drug local connectivity views (i.e., $E_{dd}$) and protein–protein local connectivity views (i.e., $E_{tt}$) are obtained similarly through multi-level information aggregation.

### 2.3. Meta-Knowledge Transfer Network

The MGACL model aims to improve the accuracy of drug–target interaction predictions by incorporating knowledge of diverse drug–target relations. In DTI prediction, it is particularly important to transfer knowledge from the characteristic information of drugs and targets to guide the learning of specific drug–target interaction patterns. To achieve this goal, we designed a meta-local connectivity knowledge transfer network to personalize and refine knowledge on both sides of the drug and target, thereby guiding drug–target interaction prediction.

#### 2.3.1. Multi-Faceted Meta-Knowledge Extraction

To obtain the interaction relationship between the auxiliary graph and the original interaction bipartite graph, we first extract meta-knowledge to preserve the important features of drugs and proteins concerning the auxiliary graph structure and the original graph structure. Specifically, the meta-knowledge extracted from the drug–local connectivity relation view and the protein–local connectivity relation view is obtained as follows:

(5) $$\begin{array}{cc} \; & M_{dd}=E_{d}\left|\right|E_{dd}\left|\right|\sum_{t\in N_{d}}e_{t},\\ \; & M_{tt}=E_{t}\left|\right|E_{tt}\left|\right|\sum_{d\in N_{t}}e_{d}, \end{array}$$

where $M_{dd}\in R^{m^{\times }3h}$ and $M_{tt}\in R^{n^{\times }3h}$ represent contextual information and generate personalized local connectivity knowledge. $E_{dd}$ and $E_{tt}$ refer to the final embeddings of the drug–local connectivity relation view and the protein–local connectivity relation view after multiple layers of a GCN. The embedding of the primitive interaction bipartite graphs captures the perceived signals of the drug- and protein-related interaction relationships. The auxiliary graph embedding represents the multifaceted properties of the drug as well as the key biological properties of the protein.

#### 2.3.2. Local Connectivity Knowledge Transfer

The meta-knowledge extracted by MGACL is used to generate a feature transfer network with a dynamic transformation matrix. The proposed meta-knowledge transfer network is

(6) $$\left\{\begin{array}{c} f_{mlp}^{1}\left(M_{dd}\right)\to W_{dd}^{M1}\\ f_{mlp}^{2}\left(M_{dd}\right)\to W_{dd}^{M2} \end{array}\right.$$

where $f_{mlp}^{1}$ and $f_{mlp}^{2}$ are meta-local connectivity knowledge learners consisting of two fully connected layers with the *LeakyReLU* activation function [24]. The function takes the meta-knowledge $M_{dd}$ as input and outputs the personalized transformation matrix $W_{dd}^{M}1\in R^{m\times h\times r}$. Both parameter tensors contain the full matrices for all drugs. Personalized node representations are generated based on the unique local connectivity knowledge of the corresponding drugs and proteins to achieve personalized knowledge transfer. The generated parameter matrices and nonlinear mapping functions are used to construct our personalized knowledge transfer network as follows:

(7) $$E_{dd}^{M}=\sigma \left(W_{dd}^{M1}W_{dd}^{M2}E_{dd}\right),$$

where σ(·) denotes the *LeakyReLU* activation function. $E_{dd}^{M}\in R^{m\times h}$ contains personalized embeddings obtained through the mapping function of the drug auxiliary graph. The personalized embedding is then used to augment the drug embedding encoded from the original interaction bipartite graph. The fusion process of drug node embedding representations is performed by the following weighted summation:

(8) $$E_{d}^{F}=\gamma_{d}*E_{d}+\left(1-\gamma_{d}\right)*\left(E_{dd}+E_{dd}^{M}\right),$$

where $\gamma_{d}\in R$ denotes the hyper-parameter that controls the weights between the original interaction graph embedding and the drug auxiliary graph embedding. The original interaction graph embedding represents the global interaction features of the drug, while the drug auxiliary graph embedding reflects the local network connectivity relationships between drugs. By adjusting the value of $\gamma_{d}\in R$, the model can balance the contributions of global and local views to obtain the final embedding representation $E_{d}^{F}$ used for downstream prediction tasks.

#### 2.3.3. Meta-Learner Optimization

The key idea of the meta-local connectivity knowledge learner is to impose regularization constraints through the meta-learning approach to enable the model to dynamically adjust its learning strategy according to different data and tasks, as well as to better handle data with complex local structures. Specifically, the consistency of each drug’s embedding with the average embedding of the local connectivity relation network is first assessed by calculating the match between the initial interaction embedding of each drug and the average embedding of the local connectivity relation network of the whole drug:

(9) $$S_{d}=\mathrm{score}\left(E_{d}^{0},\mathrm{mean}\left({\hat{A}}_{dd}E_{d}\right)\right),$$

where $E_{d}^{0}$ is the initial drug embedding of the drug–target interaction matrix, $\mathrm{mean}({\hat{A}}_{dd}E_{d})$ is the global average embedding vector after a graph convolution operation with the drug interaction embedding $E_{d}$ via the adjacency matrix ${\hat{A}}_{dd}$, and score() [25] is used to compute the matching degree between the two embeddings. Next, the matching degree between the random masked embedding of each drug and the average embedding of the whole drug–local connectivity relation network is obtained by shuffling the rows and columns of the embedding representation:

(10) $$S_{d}\text{-}\mathrm{mask}=\mathrm{score}\left(\mathrm{shuffle}\left(E_{d}^{0}\right),\mathrm{mean}\left({\hat{A}}_{dd}E_{d}\right)\right),$$

where shuffle(X) is a random mixing operation. The resulting loss function for the meta-local connectivity knowledge learner for drug embedding is obtained as follows:

(11) $$L_{d}=\mathrm{mean}\left(-\log\left(\sigma \left(S_{d}-S_{d-mask}\right)\right)\right),$$

where log(x) is the logarithmic function. Similarly, we can obtain the loss function $L_{t}$ for the meta-local connectivity knowledge learner for protein embedding. In summary, the final loss function for training the meta-local connectivity knowledge learner is

(12) $$L_{Meta}=\left(L_{d}+L_{t}\right)/2.$$

This method aims to create embedded representations that enable efficient and robust learning in complex tasks. Relying solely on rebuilding the interaction information between nodes is far from meeting the needs of downstream tasks. Next, we describe how it generates node relationships for adaptation to downstream prediction tasks through a novel contrastive learning strategy.

### 2.4. DTI Association-Aware Contrastive Learning

#### 2.4.1. Multi-View Augmentation for DTI

To enhance the representation learning capability of the MGACL framework in DTI prediction tasks and to mitigate potential problems caused by data sparsity, we designed a multi-view DTI association-aware contrastive learning paradigm. The paradigm aims to improve the robustness of heterogeneous relationship learning and pays special attention to the representation of sparsely connected nodes in DTI networks. Since drug–target interaction predictions may be biased by “popular” drugs, i.e., drugs that are connected to multiple targets tend to be predicted more easily, this may lead to biased predictions in favor of these drugs. Therefore, we pay special attention to drugs that have fewer associations in the original drug–target bipartite graph, i.e., tail drug nodes with sparse data. Our approach places special emphasis on the learning of these tail drug nodes by adjusting for contrastive loss to ensure that all drugs are effectively represented.

Specifically, we align the embedding of the auxiliary graph (i.e., $E_{dd}^{M}$ based on drug–local connectivity relations) with the embedding of the original interaction bipartite graph (i.e., $E_{d}$), so that the embedding of the auxiliary graph can be used as an effective regularization operation to influence the drug–target interaction modeling with self-supervised signals. In addition, we capture different interaction preferences between proteins by combining a multi-view meta-local connectivity knowledge transfer network with DTI association-aware contrastive learning.

#### 2.4.2. Association-Aware Contrastive Learning

Using heterogeneous graph relation learning and a meta-local connectivity knowledge transfer network, we obtain two sets of drug embeddings, $E_{dd}^{M}$ and $E_{d}$ for drugs. The embeddings are obtained by encoding the drug–target interaction data and drug-end auxiliary knowledge. We use DTI association-aware contrastive loss to coordinate graph structures from two different latent spaces to enhance the learning of drug representations for MGACL as follows:

(13) $$L_{ACL}=-\sum_{d\in V_{d}}Ass\left(d\right)\log\frac{\exp\left(\frac{s(e_{dd}^{M}+e_{dd},e_{d})}{\tau }\right)}{\sum_{d^{\prime }\in V_{d}}\exp\left(\frac{s(e_{dd}^{M}+e_{dd},e_{d}^{\prime })}{\tau }\right)},$$

where $e_{dd}^{M}\in R^{h}$ and $e_{d}\in R^{h}$ are the embedding vectors of the matrices $E_{dd}^{M}$ and $E_{d}$. s(·) denotes the similarity function. τ denotes the temperature hyper-parameter in softmax. $d^{\prime }$ denotes the negative sample, i.e., a drug node that does not have a known interaction with a specific target.

In Equation (13), we set a coefficient Ass(·) for each node to control whether the contrastive loss applies to a particular node. If the drug already has interactions with a sufficient number of proteins in the original bipartite graph, $L_{ACL}$ should endeavor to coordinate its representation in both potential spaces to prevent negative migration. We create a variant Sigmoid function for Ass(·):

(14) $$Ass\left(d\right)=1-\frac{r}{r+\exp\left(\frac{\sum_{t\in V_{t}}\varepsilon_{dt}}{s}\right)},d\in V_{d},$$

where $\sum_{t\in V_{t}}\varepsilon_{dt}$ refers to the number of interactions of the drug $d\in V_{d}$ overall with different targets, called its degree on $G_{dt}$, and *r* is a hyper-parameter controlling the rate of increase in Ass(d) to approximately 1. The specific modulation of Ass(·) can be divided into the following three phases, where the drug’s relevance perception coefficient is close to zero during the tail phase of the data sparsity when the effect of contrastive learning is almost stagnant. However, in the data-rich middle and head stages, the learned representation of the drug becomes more accurate and confident as the drug’s interactions with different targets gradually increase. Therefore, in these phases, the drug’s correlation perception coefficient is gradually increased to 1 to encourage contrastive learning and optimize the prediction of drug–target interactions.

### 2.5. Optimization of MGACL

To optimize our model for application in DTI prediction tasks, we use a pairwise loss function similar to Bayesian Personalized Ranking (BPR) [26]. In this case, each training sample consists of a target *t*, a positive sample drug $d^{+}$ with known interactions, and a negative sample drug $d^{-}$ with unknown interactions. For each training sample, we aim to maximize the interaction prediction score between the target and the positive drug while minimizing the prediction score with the negative drug:

(15) $$J\left(\theta \right)=L_{bpr}=\sum_{(t,d^{+},d^{-})\in O}-\ln\left(\sigma ({\hat{y}}_{t,d^{+}}-{\hat{y}}_{t,d^{-}})\right)+\lambda {\left\|\Theta \right\|}^{2},$$

where ln(·) and σ(·) denote the logarithmic and *sigmoid* functions, respectively. λ denotes the hyper-parameter that determines the weight of the regularization term. The meta-local connectivity knowledge learner is updated on both $L_{Meta}$ and $L_{ACL}$ with the following negative sample generation loss:

(16) $$F\left(\theta \right)=L_{\mathrm{Meta}}+\mu L_{ACL},$$

where μ is the hyper-parameter that controls the weights of the DTI association-aware contrastive learning. In using a hybrid optimization strategy combining prediction loss and negative sample generation loss, the overall training loss is as follows:

(17) $$\min_{\theta }J\left(\theta \right)+\alpha *F\left(\theta \right),$$

where α regulates the important hyper-parameters of the corresponding loss function. Using the hybrid optimization strategy described above allows the model to optimize at multiple levels simultaneously by minimizing the model loss, improving the model’s ability to focus on optimizing the prediction ranking task as well as its ability to capture knowledge about personalization, while allowing the model to better discriminate between positive and negative samples.

## 3. Results

In this section, we conduct a detailed evaluation of the MGACL model and analyze its effectiveness by comparing it with baseline methods. In addition, we further analyze the key modules and the robustness of models in different schemes.

### 3.1. Experimental Settings

#### 3.1.1. Datasets

To extensively evaluate the performance of the model, five datasets were used in the experiments: our own powerful multi-source heterogeneous information dataset, Luo’s DTIdata [27], and three datasets collected by Yamanishi’s DTIdata [28], i.e., G-protein-coupled receiver (GPCR), ion channels (IC), and enzymes. Luo’s DTIdata, as one of the most commonly used benchmark datasets in the field of DTI prediction, has been widely recognized by researchers for its soundness and standardization of establishment. However, drug-target interactions are constantly being discovered and identified over time, and to avoid relying only on data that may be outdated, we used the same data collection strategy as Luo’s DTIdata for the dataset establishment. Specifically, the drug nodes, protein nodes, disease nodes, and side effect nodes involved in our dataset were obtained from the DrugBank database (Version 5.1.10) [29], the Uniprot database (Release 2023.05) [30], the Comparative Toxicogenomics Database (Release 2023.10) [31], and the SIDER database (Version 4.1) [32]. This approach ensures that our datasets are of high scientific and practical value in terms of coverage and timeliness, providing a solid foundation for studying drug–target interactions. Due to the small size of the nuclear receptor dataset, only the remaining three datasets in Yamanishi’s DTIdata were used in this study. The data statistics of the different datasets are shown in Table 1. These three datasets, varying in size from large to medium to small, offer a more comprehensive assessment of the model.

#### 3.1.2. Baselines

To verify the effectiveness of MGACL in the field of DTI prediction, we compared it with the following 12 methods, which can be classified into four classes: (1) the most representative heterogeneous network-based methods in the field of DTI prediction, i.e., DTINet [27], NeoDTI [33], GCN-DTI [34], and EEG-DTI [25]; (2) the machine learning-based novel feature fusion methods, i.e., ${\mathrm{FGS}}_{\mathrm{GRMF}}$ [7], MSI-DTI [35], and HMSA-DTI [36]; (3) meta-path-based/adaptive meta-graph-based methods, i.e., IMCHGAN [37], HampDTI [38], and AMGDTI [39]; and (4) contrastive learning-based methods, i.e., SGCL-DTI [13] and SHGCL-DTI [40]. Please note that since HampDTI [38] and AMGDTI [39] design meta-path/meta-graphs based on multiple relational heterogeneous networks only, they are not applicable to *Yamanishi’s DTIdata*. We introduce all baseline models here:

- DTINet [27]: This method uses a random walk algorithm and a dimensionality reduction scheme to derive low-dimensional feature vectors of nodes.
- NeoDTI [33]: This method extracts complex hidden features of various types of nodes through information passing and aggregation operations.
- GCN-DTI [34]: This method constructs a drug- and protein-based network, and considers the DTI prediction problem as a node classification problem.
- EEG-DTI [25]: This method makes predictions by learning low-dimensional representations of different nodes.
- ${\mathrm{FGS}}_{\mathrm{GRMF}}$ [7]: This method proposes a fine-grained selective similarity integration approach (FGS). Predictions are made by integrating FGS into the machine learning model [41].
- IMCHGAN [37]: This method proposes a two-level graph attention network (GAT) to learn node latent features from the heterogeneous network and uses inductive matrix completion to predict DTIs.
- HampDTI [38]: This method uses a learnable attention mechanism to automatically extract useful meta-paths, learning multi-channel node embeddings via a GCN without relying on domain knowledge.
- AMGDTI [39]: This method constructs adaptive meta-graphs for drugs and proteins separately using a GCN to extract potential features.
- SGCL-DTI [13]: This method proposes a new positive and negative sample selection strategy to guide model optimization in a supervised manner.
- SHGCL-DTI [40]: This method proposes an auxiliary graph contrastive learning task for DTI prediction.
- MSI-DTI [35]: This method obtains feature representations from different views by integrating biometric features and knowledge graph representations from multiple sources of information.
- HMSA-DTI [36]: This method utilizes a layered multimodal self-attention mechanism to achieve deep fusion of multimodal features of drugs and proteins, thereby capturing the interactions between drugs and proteins.

#### 3.1.3. Hyper-Parameter Settings

We implemented our MGACL model in PyTorch and optimized its parameters using Adam. The batch size and learning rate are searched from [4,8,16,32,64,128] and [$10^{-4}$,$5\times 10^{-4}$,$10^{-3}$,$5\times 10^{-3}$,$10^{-2}$] for grid search, respectively. The embedding size was scaled in the range of [16,32,64,128,256]. To prevent overfitting, we added $L_{2}$ regularization coefficients, which are scaled from the range [0.01,0.015,0.02,0.03,0.05,0.1]. When the performance on the validation set did not change significantly for five consecutive epochs, early stopping is used to select the best model. The number of GNN layers is selected from [1,2,3]. In addition, the coefficient of contrastive loss μ is selected from [0.5,0.8,1.0,1.2,1.5]. The temperature parameters are chosen from [0.1,0.3,0.5,0.6]. We used the published source code and recommended parameters from their respective papers for all baselines to ensure optimal results.

Following [27], we excluded isolated nodes in the network. In our experiments, we considered drug–target pairs with known interactions as positive samples and the remaining drug–target pairs as negative samples. To more objectively assess the performance of MGACL, a five-fold cross-validation was performed on five datasets, and the average performance is reported. The experiments divided the datasets in an 8:1:1 ratio, and for each positive sample, the same number of negative samples were randomly sampled. Following the setup of most DTI prediction experiments, we chose to evaluate the performance of our model using the area under the curve (AUC) and the area under the precision–recall curve (AUPRC).

### 3.2. Performance Comparison

We summarize the performance of different models in terms of the AUC and AUPRC based on their performance on different datasets in Table 2. It shows that MGACL outperforms other methods in all evaluation metrics. In addition, we have the following observations:

- Our MGACL consistently achieves significant performance improvements over the most representative techniques based on heterogeneous networks. We believe that these improvements all stem from the design of the meta-knowledge transfer network combined with the association-aware contrastive learning paradigm: (1) MGACL allows node representations to efficiently transfer knowledge between heterogeneous relationships to aid in DTI prediction; (2) adaptive DTI association-aware contrastive learning significantly improves prediction performance by leveraging self-supervised signals between heterogeneous relationship views to enhance feature extraction.
- Compared to the fine-grained feature fusion method ${\mathrm{FGS}}_{\mathrm{GRMF}}$, which is based on machine learning, MGACL has made great progress, especially in the AUPRC performance. The AUPRC provides a more objective evaluation of highly skewed data. The proposed novel contrastive learning strategy utilizes self-supervised signals and can learn effective feature representations from highly skewed data to extract deeper, abstract features, thus improving the feature learning capability of the model.
- Although meta-path-based/adaptive meta-graph-based approaches obtain good performance gains and are more flexible than fixed meta-path methods relying on domain expert knowledge definitions, they cannot effectively learn multiplex relational signals between multiple types of nodes in a multiplex heterogeneous network. For MGACL, by effectively integrating multiple knowledge sources, meta-knowledge transfer networks can capture complex data relations more comprehensively, thus providing more robust performance across different tasks and datasets.
- The superior performance of SGCL-DTI and SHGCL-DTI justifies the use of self-supervised learning to enhance drug–protein interaction coding. The performance gains shown by our MGACL when comparing the above methods validate that the node representations learned from the tilted graph structure contain biased information, and considering how to reduce negative migration further improves the performance.

### 3.3. Ablation Study

We performed ablation studies to validate the effectiveness of each module in our MGACL as described below:

- w/o-meta: MGACL does not contain meta-networks that allow for personalized knowledge transfer in contrastive learning across relational views.
- w/o-dd: In this variant, we do not include the drug–local connectivity relational graph $G_{dd}$ to capture knowledge-aware dependencies between drugs to guide the learning process for drug–target interaction prediction.
- w/o-tt: In this variant, we do not include the target local connectivity relational map $G_{tt}$ to consider biological or functional interrelationships between targets to help encode target–drug interaction patterns.
- ${\mathrm{MGACL}}_{N}$: It indicates that we have no contrastive learning between the learned auxiliary map and the original map.
- ${\mathrm{MGACL}}_{P}$: It indicates that we implement contrastive learning on the graph of MGACL but do not consider DTI associations (i.e., deleting Ass(·) in Equation (13)).

The performances of the MGACL model and its variants after five-fold cross-validation are shown in Table 3. w/o-meta performs worse than MGACL on different datasets. This result is consistent with our hypothesis that transferring and extracting local connectivity relationships between nodes in different graph structures unbiasedly helps to learn the embedding representation of nodes better. MGACL shows a better performance relative to both w/o-dd and w/o-tt, implying that it is necessary to incorporate heterogeneous auxiliary information into the model to guide the drug–protein interaction coding. Finally, MGACL and ${\mathrm{MGACL}}_{P}$ achieve significant improvements on all datasets compared to ${\mathrm{MGACL}}_{N}$, which validates the alignment between the learned auxiliary and original graphs, which minimizes the differences between the auxiliary and original graph structures. However, ${\mathrm{MGACL}}_{P}$ performs worse than MGACL when we align the two views of sparse data nodes. This indicates that the representation of these nodes in the original graph structure is uncertain and disrupts the learning of the auxiliary graph, validating the necessity of the DTI association-aware coefficient Ass(·) in $L_{ACL}$.

### 3.4. Performance Variations in Data Imbalance Scenarios

Drug–target interaction prediction is critical for new drug discovery and drug repositioning. One of the challenges in this area is how to deal with the inherent imbalance of experimental data: The number of known drug–target interactions (positive samples) is usually much smaller than the number of unknown or absent interactions (negative samples). In the real world, this imbalance is pervasive, and choosing different positive and negative sample ratios is particularly important for assessing model performance. To simulate the real-world situation where there are usually only a few known interactions in the data, we divided the data into three separate groups according to different numbers of negative samples for cross-validation:

- Group G1: Balanced positive and negative samples in the test data.
- Group G2: The negative samples in the test data are ten times the positive samples.
- Group G3: The negative samples in the test data are all remaining non-interacting pairs not present in the training data.

**Figure 2:**
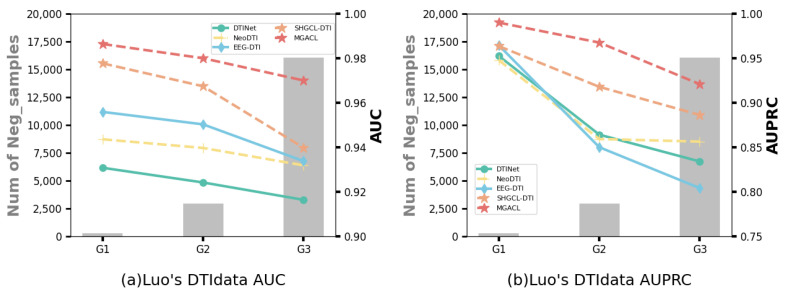
Performance comparison concerning different data imbalance scenarios on *Luo’s DTIdata*. The lines display the performance of each method, shown on the right side of the y-axis. Bars show the number of negative samples for each negative sampling strategy, with their values on the left side of the y-axis. (**a**,**b**), respectively, show the changes in AUC and AUPRC of different methods in different scenarios.

The known DTIs (i.e., positive samples) account for only 10% and 1.8% of the entire dataset after applying the unbalanced settings of Group G2 and Group G3, respectively, to *Luo’s DTIdata*. The results of the performance comparison between MGACL and several baselines on *Luo’s DTIdata* are shown in Figure 2. The performance of each method is represented by lines, and their values are displayed on the right side of the y-axis. A bar is used to represent the number of negative samples in the test set for each negative sampling strategy, and their values are displayed on the left side of the y-axis. Our method demonstrates superior performance with varying degrees of skewed data, especially in the AUPRC performance, which is more objective for evaluating unbalanced data. The improvement of MGACL may stem from adaptive enhancement through customized association-aware contrastive learning. This approach effectively captures the complex interactions between drugs and targets as well as their respective properties. As a result, in our experiments, MGACL demonstrated excellent performance even when the data were sparse and the positive and negative samples were severely unbalanced. This demonstrates that MGACL can adapt to the common data imbalance problem in DTI prediction and still has superior robustness and prediction accuracy in such scenarios. More performance evaluation results under different splitting strategies are presented in Supplementary Materials S1.

### 3.5. Generalization Performance Evaluation

To further evaluate the generalization performance of MGACL, we used holdout validation for MGACL and several baselines. Specifically, a brand new independent test set was set up to ensure that it was untouched during model training and tuning. A potential drawback of holdout validation is that the results may be affected by specific data segmentation methods. To mitigate this issue, we performed ten holdout validations, each using a different random seed for data segmentation. We ultimately report the average of the ten results for a more stable and reliable performance evaluation. The final results are shown in Table 4. MGACL outperforms other baseline methods in terms of AUC and AUPRC, which is consistent with the results obtained from the five-fold cross-validation. This shows that MGACL still has good generalization performance for brand-new and unseen data.

### 3.6. Hyper-Parameter Analysis

**Figure 3:**
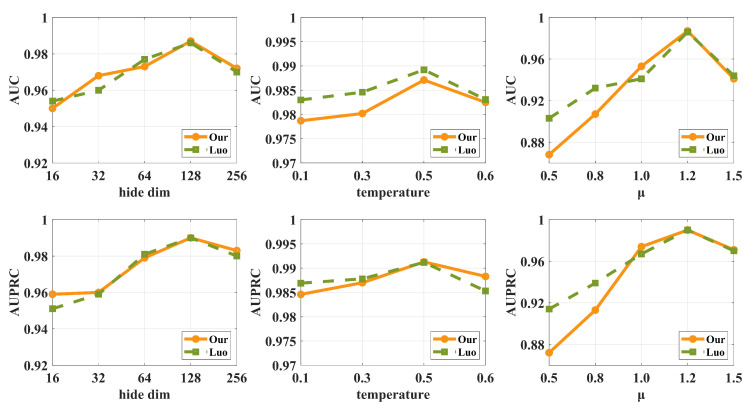
Hyper-parameter study on two datasets.

For MGACL, further parameter sensitivity analyses are performed to explore the effects of the hidden layer dimension *h*, temperature parameter τ, and the hyper-parameter μ, respectively. The results are shown in Figure 3, and we obtained the following conclusions:

- The selection of the hidden layer dimension *h* ranges from 16 to 256. We observe that the model reaches its optimal performance when *h* = 128 and then starts to decline. Therefore, increasing the embedding dimension appropriately can improve the model performance. Still, there may be a risk of overfitting when the model is too complex and the embedding dimensions are too high.
- For the value of τ, we observe that the model performance is optimal when the τ is 0.5, while the performance decreases at 0.6. This suggests that an appropriate temperature parameter allows the model to identify the correct category accurately, but too large a temperature parameter may cause the model to start focusing too much on the noise in the training data or features that are not important, leading to performance degradation.
- For the contrastive learning hyper-parameter, we observe that the model reaches its optimal performance when μ is 1.2, while there is a decreasing trend at 1.5. This suggests that the decrease in performance when μ is increased to 1.5 may be a sign of over-tuning. This could mean that higher values of μ cause the model to over-adapt to the specific features of the current dataset, thus affecting its generalization ability.

### 3.7. Case Study

**Figure 4:**
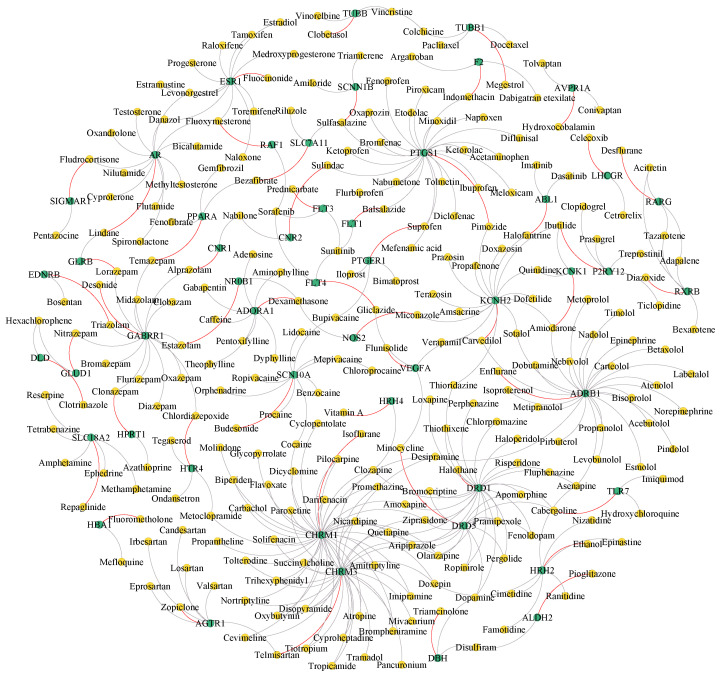
Visualization of the overall drug–target interaction network involving the top 50 MGACL predictions. Targets and drugs are shown in green circles and yellow circles, respectively. Gray edges denote known interactions, and red edges indicate predicted novel interactions.

We used the entire heterogeneous network (where each target has at least one drug with known interactions) as training data to predict and output a list of the top 50 predictions, as shown in Figure 4.

Next, the top 10 drug–target pairs from the top 50 prediction list were selected for further study in our case study. As shown in Table 5, 9 of the top 10 drug–target pairs have conclusive evidence in the database, comparable to other similarity integration methods, demonstrating MGACL’s reliability in predicting potential DTIs, findings that are crucial for understanding intermolecular mechanisms of action as well as for further drug design. Meanwhile, we found an interesting phenomenon: the eighth prediction in Table 5 ranked high for prediction but did not receive relevant experimental support in the literature. To verify the validity of the unknown predictions, we downloaded the amino acid sequences of the proteins from Uniport [30], performed homology modeling and docking simulations of the proteins using SWISS-MODEL [42] and AutoDock [43], and finally obtained 2D and 3D visualization results using PyMOL [44].

**Figure 5:**
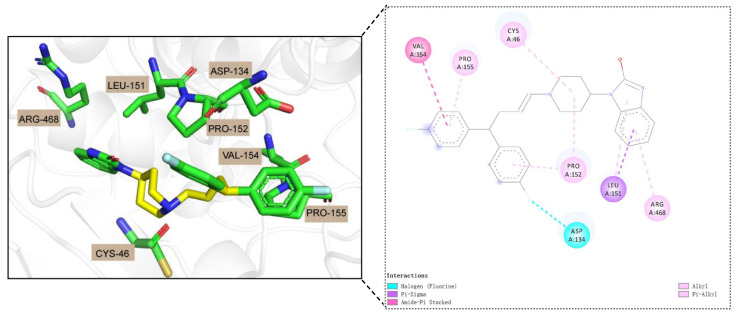
The 2D and 3D visualizations of docked poses between Pimozide and PTGS1. Among them, green represents carbon atoms, blue represents nitrogen atoms, and red represents oxygen atoms.

PTGS1 is one of the main targets of action of non-steroidal anti-inflammatory drugs (NSAIDs), which act as anti-inflammatory and analgesic agents by inhibiting PTGS1 to reduce prostaglandin production [45]. For Pimozide, although it is mainly considered a psychiatric drug for the treatment of psychiatric disorders [46], its binding to cyclooxygenase 1 (PTGS1) can reach −10.7 kcal/mol based on our predictive modeling, implying that there may be a strong interaction between the two, which deserves further experimental validation. The corresponding molecular docking studies are shown in Figure 5, where Pimozide can dock with the structure of PTGS1, displaying a specific binding mode. In particular, Pimozide adapts to the active site of PTGS1, showing three main intermolecular interactions: the Pi-Sigma, Amide-Pi Stacked, and Halogen bonds. These interactions demonstrate the high-affinity binding energy predicted for Pimozide with PTGS1 and provide evidence at the molecular level for understanding the potential anti-inflammatory mechanism of action of the drug. The Pi-Sigma and Amide-Pi Stacked interactions point to the importance of the aromatic ring in stabilizing the drug–protein complex, whereas the Halogen bonds further stabilize this complex. These findings provide new insights into the potential of Pimozide as a COX inhibitor and may contribute to the development of novel anti-inflammatory drugs that may have a different side effect profile than existing NSAIDs. Therefore, our research provides new strategies for future drug discovery and drug design, and emphasizes the value of utilizing existing drugs for new indications. More molecular docking results are presented in Supplementary Materials S2.

## 4. Conclusions

Target discovery is an important task in developing new drugs, elucidating molecular mechanisms of drugs and searching for indications. In this paper, we propose a novel framework, MGACL, that utilizes an adaptive enhanced personalized meta-knowledge transfer network to transfer heterogeneous auxiliary information from different nodes. It has been demonstrated that this method can comprehensively analyze and extract massive amounts of multi-source heterogeneous data and creatively use the contrastive learning strategy of DTI association perception to reduce the negative transfer caused by low-frequency drug representation, thereby ensuring data accuracy while avoiding information omission. Through the combination of MGACL prediction and practical application, we identified target proteins for some traditional drugs, which exhibit high scores in docking experiments. The accuracy of the model prediction is about 3% better than that in existing research based on the AUC and AUPRC. Our research provides a valuable predictive tool for the discovery of new targets for active compounds or traditional drugs, which will drive the development process of new drugs.

## Figures and Tables

**Table 1:** Descriptive statistics of the datasets.

	Our DTIdata	Luo’s DTIdata	Yamanishi’s DTIdata		
			GPCR	Enzyme	IC
Drug	1269	708	223	445	210
Protein	1615	1512	95	664	204
Known Interaction	5225	1923	635	2926	1476
Sparsity	99.745%	99.820%	97.003%	99.010%	96.555%

**Table 2:** Performance comparison of all methods on different datasets in terms of AUC and AUPRC.

Model	Our DTIdata		Luo’s DTIdata		Yamanishi’s DTIdata					
	AUC	AUPRC	AUC	AUPRC	GPCR		Enzyme		IC	
					AUC	AUPRC	AUC	AUPRC	AUC	AUPRC
DTINet	0.8634	0.8849	0.9308	0.9526	0.8833	0.8789	0.9338	0.9518	0.9139	0.9089
NeoDTI	0.9218	0.9346	0.9436	0.9478	0.9258	0.9077	0.9874	0.9829	0.9431	0.9567
GCN-DTI	0.8859	0.8962	0.9192	0.8764	0.9183	0.9046	0.9712	0.9834	0.9741	0.9759
EEG-DTI	0.9036	0.8973	0.9559	0.9645	0.961	0.9615	0.9834	0.9858	0.9841	0.9832
${\mathrm{FGS}}_{\mathrm{GRMF}}$	0.9041	0.8262	0.9397	0.8082	0.9134	0.8347	0.9546	0.8573	0.9357	0.8489
IMCHGAN	0.9329	0.9546	0.9563	0.9872	0.9485	0.9479	0.9637	0.9518	0.9542	0.9483
HampDTI	0.9236	0.9149	0.9279	0.9263	/	/	/	/	/	/
AMGDTI	0.936	0.9441	0.978	0.979	/	/	/	/	/	/
SHGCL	0.9563	0.9742	0.9577	0.9636	0.9531	0.9458	0.9836	0.9751	0.9863	0.9849
MSI-DTI	0.9632	0.9769	0.9689	0.9746	0.9601	0.9589	0.9852	0.9760	0.9869	0.9851
SGCL *	/	/	0.9771	0.9768	0.9741	0.9812	0.9892	0.9895	0.9857	0.9852
HMSA-DTI	0.9794	0.9770	0.9799	0.9809	0.9772	0.9839	0.9890	0.9805	0.9877	0.9864
**MGACL**	**0.9871**	**0.9907**	**0.9892**	**0.9912**	**0.9807**	**0.9845**	**0.9939**	**0.995**	**0.9941**	**0.9954**

“/” denotes that the model is not applicable to the dataset; “*” denotes the experimental results of the literature we cited; boldface denotes optimal performance.

**Table 3:** Ablation study on key components of MGACL.

Model	Our DTIdata		Luo’s DTIdata	
	AUC	UPRC	AUC	AUPRC
w/o-meta	0.9670	0.9767	0.9653	0.9753
w/o-dd	0.9749	0.9772	0.9755	0.9864
w/o-tt	0.9642	0.9749	0.9715	0.9802
$MGACL_{N}$	0.9704	0.9796	0.9726	0.9807
$MGACL_{P}$	0.9758	0.9824	0.9784	0.9846
MGACL	0.9871	0.9907	0.9892	0.9912

**Table 4:** Holdout validation on Luo’s DTIdata.

Model	AUC	AUPRC
DTINet	0.9167	0.9105
NeoDTI	0.9357	0.9302
HampDTI	0.9209	0.8972
**MGACL**	**0.9713**	**0.9682**

*Note*: The bold values indicate the best performance for each metric.

**Table 5:** Top 10 predicted results discovered by MGACL from Luo’s DTIdata.

Rank	Drug ID	Drug Name	Target ID	Target Name	Evidence
1	DB00988	Dopamine	P09172	DBH	DrugBank
2	DB01223	Aminophylline	P30542	ADORA1	DrugBank
3	DB00619	Imatinib	P00519	ABL1	DrugBank
4	DB00988	Dopamine	P21728	DRD1	DrugBank
5	DB01268	Sunitinib	P35916	FLT4	DrugBank
6	DB00384	Triamterene	P51168	SCNN1B	DrugBank
7	DB01248	Docetaxel	Q9H4B7	TUBB1	DrugBank
8	DB01100	Pimozide	P23219	PTGS1	Unknown
9	DB01268	Sunitinib	P17948	FLT1	DrugBank
10	DB00740	Riluzole	Q9UPY5	SLC7A11	DrugBank

## Data Availability

Luo’s dataset is available on GitHub (https://github.com/luoyunan/DTINet, accessed on 31 July 2024). Yamanishi’s dataset is available at https://members.cbio.mines-paristech.fr/~yyamanishi/pharmaco/, accessed on 31 July 2024.

## Supplementary Materials

The following supporting information can be downloaded at: https://www.mdpi.com/article/10.3390/biom14101267/s1, Figure S1: Performance evaluation under different splitting strategies; Figure S2: The 2D and 3D visualization of docked pose. Ref. [47] is cited in the Supplementary Materials.
