# Supplementary material for: MGACL: Prediction Drug–Protein Interaction Based on Meta-Graph Association-Aware Contrastive Learning
Source: Biomolecules. 2024 Oct 8;14(10):1267. doi: 10.3390/biom14101267 (PMC11505808; doi:10.3390/biom14101267)
Supplement: Supplementary file 1 [file biomolecules-14-01267-s001.zip › biomolecules-3166147-supplementary.pdf]

Supplementary Materials

S1. Performance evaluation under different splitting strategies

Without introducing unknown relationships, deep learning-based models have achieved promising performance. However, there are some hidden biases in datasets, and high-precision results may be due to biases and overfitting. Therefore, we further use the cold pair splitting strategy to evaluate the model, to alleviate the overly optimistic performance estimation under random splitting caused by data bias. This cold pair segmentation strategy ensures that not all test drugs and proteins are observed during the training process, so the prediction of test data cannot rely solely on the characteristics of known drugs or proteins.

We employ the following strategies to partition the dataset for performance evaluation under different splitting approaches. Group 1 (G1): Randomly removing 5% and 10% of drugs from the training set, and reallocating the corresponding drug-target interaction (DTI) pairs to the validation and test sets; Group 2 (G3): Randomly removing 5% and 10% of proteins from the training set, and reallocating the associated DTI pairs to the validation and test sets; and Group 3 (G3): Randomly assigning 5% and 10% of DTI pairs to the validation and test sets, and removing all related drugs and proteins from the training set. As shown in Figure S1, while the performance of all models significantly declined before the split, MGACL still achieved the best performance compared to other state-of-the-art deep learning baselines.

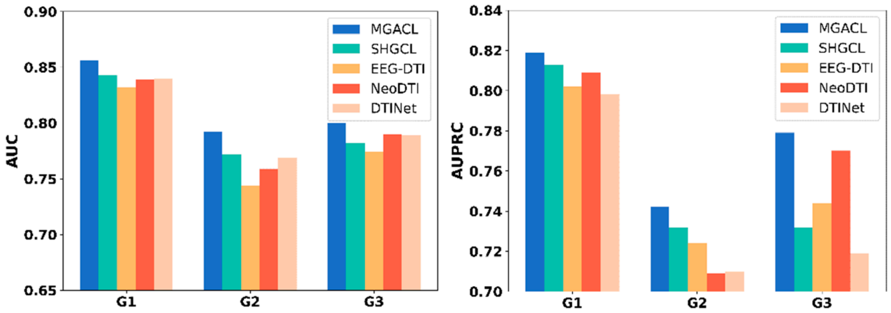

Figure S1. Performance evaluation under different splitting strategies

S2. Case study

Deep learning is well known as a black-box model, primarily focusing on whether the feature extractor meets the desired mapping requirements for the input. However, identifying which features are important and which are redundant is often challenging. The lack of interpretability

frequently limits the application of DL models.

In existing studies, such as [1], [2], and [3], molecular docking is used to verify the reliability of prediction results. Referring to these advanced studies, we additionally conduct molecular docking experiments on the top-scoring predicted pairs that lacked validation from known information, and the combination scores and their docking models are shown in Figure S2. More than 80% of these prediction pairs have binding scores higher than -7kcal\mol, and the docking results indicate that the potential interactions we predicted are reliable.

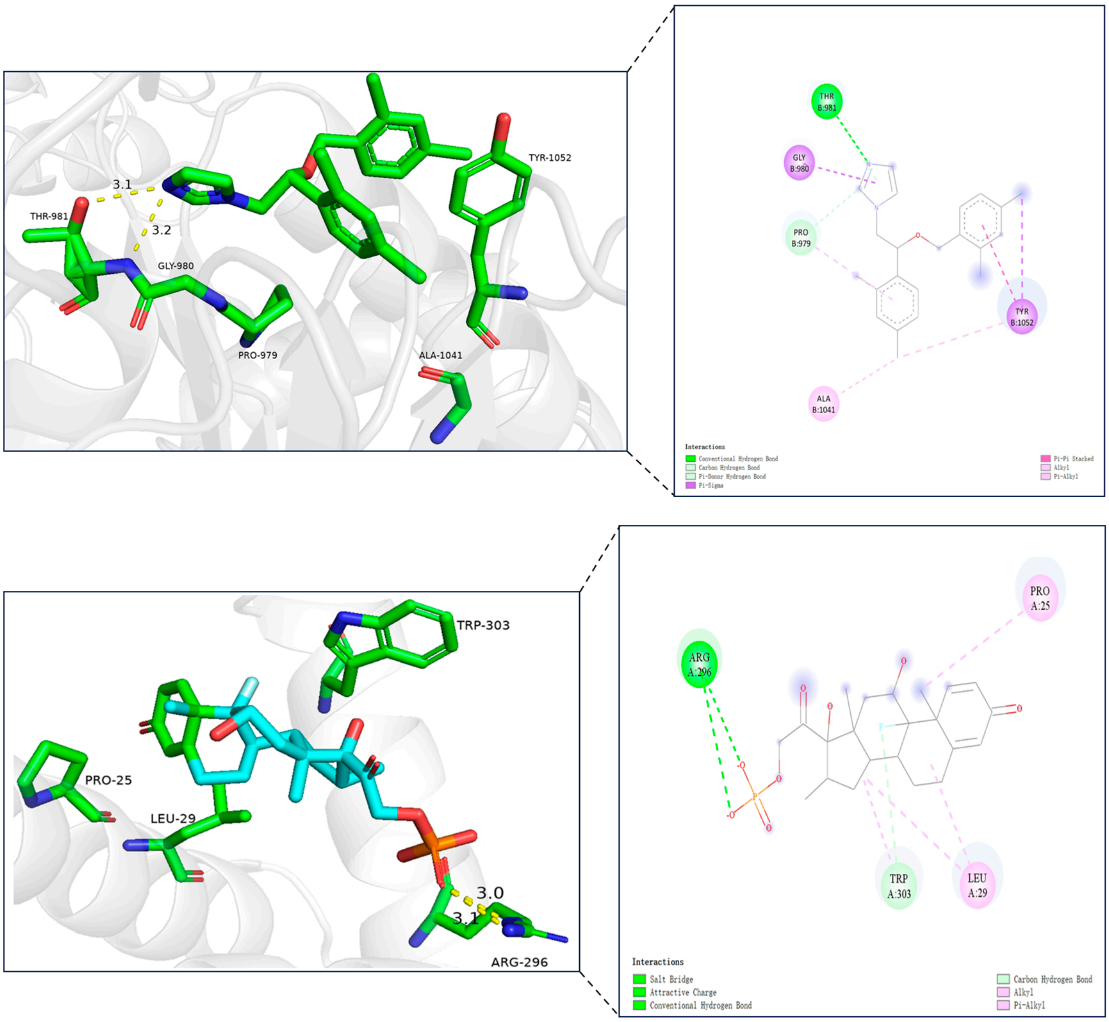

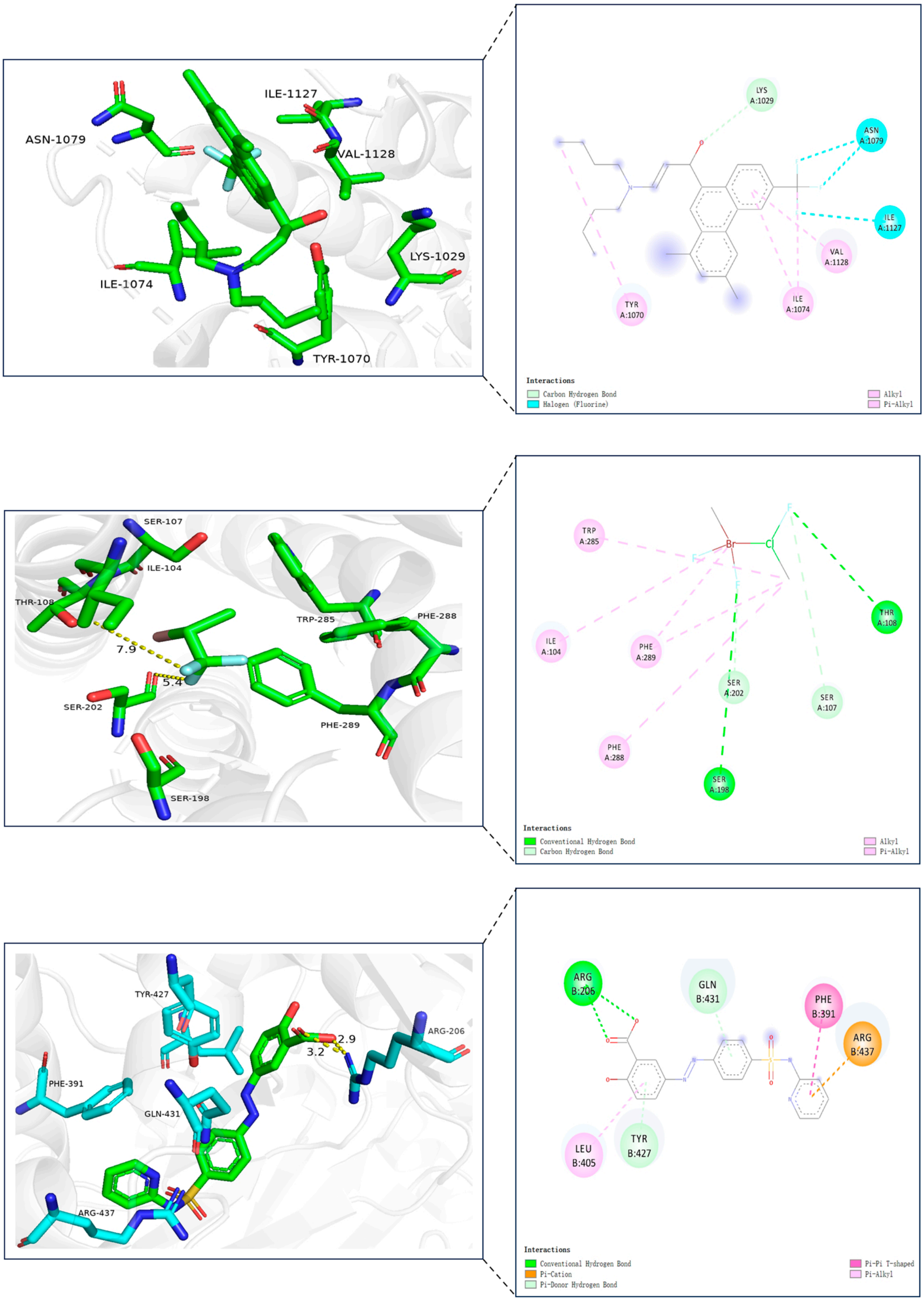

Figure S2. The 2D and 3D visualization of docked pose.

[1] Bai, Peizhen, et al. "Interpretable bilinear attention network with domain adaptation improves drug–target prediction." *Nature Machine Intelligence* 5.2 (2023): 126-136.

[2] Liu, Bin, et al. "Fine-grained selective similarity integration for drug–target interaction prediction." *Briefings in Bioinformatics* 24.2 (2023).

[3] Su, Yansen, et al. "AMGDTI: drug–target interaction prediction based on adaptive meta-graph learning in heterogeneous network." *Briefings in Bioinformatics* 25.1 (2024).
